# Influence of Inflammatory Polyarthritis on Quantitative Heel Ultrasound Measurements

**DOI:** 10.1186/1471-2474-13-133

**Published:** 2012-07-26

**Authors:** Stephen R Pye, Tarnya Marshall, Karl Gaffney, Robert Luben, Kay-Tee Khaw, Alan J Silman, Deborah PM Symmons, Terence W O’Neill

**Affiliations:** 1 Arthritis Research UK Epidemiology Unit, The University of Manchester, Manchester Academic Health Science Centre, Manchester, UK; 2 Norfolk and Norwich University Hospitals Trust, Norwich, UK; 3 Strangeways Research Laboratory, University of Cambridge, Cambridge, UK; 4 Current address: Arthritis Research UK, Chesterfield, UK

## Abstract

**Background:**

There are few data concerning the impact of inflammatory polyarthritis (IP) on quantitative heel ultrasound (QUS) measurements. The aims of this analysis were i) to determine the influence of IP on QUS measurements at the heel and, ii) among those with IP to determine the influence of disease related factors on these measurements.

**Methods:**

Men and women aged 16 years and over with recent onset IP were recruited to the Norfolk Arthritis Register (NOAR). Individuals with an onset of joint symptoms between 1989 and 1999 were included in this analysis. At the baseline visit subjects underwent a standardised interview and clinical examination with blood taken for rheumatoid factor. A population-based prospective study of chronic disease (EPIC-Norfolk) independently recruited men and women aged 40 to 79 years from the same geographic area between 1993 and 1997. At a follow up assessment between 1998 and 2000 subjects in EPIC-Norfolk were invited to have quantitative ultrasound measurements of the heel (CUBA-Clinical) performed. We compared speed of sound (SOS) and broadband ultrasound attenuation (BUA), in those subjects recruited to NOAR who had ultrasound measurements performed (as part of EPIC-Norfolk) subsequent to the onset of joint symptoms with a group of age and sex matched non-IP controls who had participated in EPIC-Norfolk. Fixed effect linear regression was used to explore the influence of IP on the heel ultrasound parameters (SOS and BUA) so the association could be quantified as the mean difference in BUA and SOS between cases and controls. In those with IP, linear regression was used to examine the association between these parameters and disease related factors.

**Results:**

139 men and women with IP and 278 controls (mean age 63.2 years) were studied. Among those with IP, mean BUA was 76.3 dB/MHz and SOS 1621.8 m/s. SOS was lower among those with IP than the controls (difference = −10.0; 95% confidence interval (CI) –17.4, -2.6) though BUA was similar (difference = −1.2; 95% CI −4.5, +2.1). The difference in SOS persisted after adjusting for body mass index and steroid use. Among those with IP, disease activity as determined by the number of swollen joints at baseline, was associated with a lower SOS. In addition SOS was lower in the subgroup that satisfied the 1987 ACR criteria. By contrast, disease duration, steroid use and HAQ score were not associated with either BUA or SOS.

**Conclusions:**

In this general population derived cohort of individuals with inflammatory polyarthritis there is evidence from ultrasound of a potentially adverse effect on the skeleton. The effect appears more marked in those with active disease.

## Background

Rheumatoid arthritis (RA) is associated with an increased risk of both hip and vertebral fracture [1-8]. Data from clinic based and population studies suggest that bone mass is reduced in subjects with RA compared with non-RA controls which may in part explain this increased risk [9-14]. Quantitative heel ultrasound measurements including speed of sound (SOS) and broadband attenuation (BUA) have been shown to be associated with risk of spine and non spine fracture in men and women [15-21]. There is some evidence that patients with RA have a reduction in heel ultrasound parameters compared to non RA controls [22-30]. Most of these studies though include patients who were recruited from secondary care and therefore likely to have more severe and longer duration of disease. Furthermore there are few data in the literature concerning the influence of disease activity related factors on heel ultrasound parameters in individuals with inflammatory arthritis [22,24-29,31]. We therefore studied a group of men and women recruited to the Norfolk Arthritis Register, a unique primary care based cohort of men and women with new onset inflammatory polyarthritis in which information about disease activity is recorded in a standardised fashion and an appropriately derived comparison group. The aim of this analysis was i) to determine the influence of IP on SOS and BUA as measured using heel ultrasound and, ii) among those with IP to determine the influence of disease related factors on these parameters.

## Methods

### Design

Subjects with IP and non-IP controls were recruited from the same population source: the Norfolk cohort of the European Prospective Investigation of Cancer (EPIC-Norfolk). Cases were individuals with inflammatory polyarthritis (IP) notified to the Norfolk Arthritis Register (NOAR) and who had also subsequently participated in EPIC. Two controls (age and sex matched) were selected for each case, being subjects who had been recruited to EPIC-Norfolk and who were not registered with NOAR. To determine the influence of IP on SOS and BUA, we compared these measurements between the cases and matched controls. To determine the influence of disease activity variables on these parameters we restricted our analysis to just the subjects with IP. Both EPIC-Norfolk and NOAR were in compliance with the Helsinki Declaration and approved by the ethics committee of the Norwich District Health Authority.

### Norfolk Arthritis Register (NOAR)

NOAR is a primary care based inception cohort of adults aged 16 years and over, registered with a local GP, with early IP in the former Norfolk Health Authority (UK). Subjects are included in the register if they had 2 or more swollen joints for a period of at least 4 weeks, with onset since January 1, 1989. Detailed methods for the study have been published previously [32]. The current analysis includes subjects with an onset of symptoms before 2000. At baseline, subjects completed an interviewer administered questionnaire and Health Assessment Questionnaire [33]. They were examined by a trained nurse for the presence of tender and swollen joints (51 joints), and had blood taken for rheumatoid factor measurement. As explained elsewhere, subjects had x-rays of the hands and feet performed if they satisfied the ACR criteria for RA or if the presence of erosions would make them satisfy these criteria [34]. The ACR criteria were applied at the baseline visit.

### European Prospective Investigation of Cancer (EPIC-Norfolk)

Subjects were recruited in Norfolk, UK for participation in the European Prospective Investigation of Cancer – a large multicentre population based study with the primary aim of establishing the relationship between diet and the risk of developing cancer. In Norfolk, approximately 25,000 men and women aged 40–79 years were recruited between 1993 and 1997. Subjects completed a health questionnaire which included information about steroid therapy for 3 or more months. Height and weight were measured in light clothing without shoes. Body mass index (BMI) was calculated using measurements made at the baseline EPIC visit by dividing weight (Kg) by height squared (m^2^). Detailed methods have been published previously [35].

### Calcaneal ultrasound

In 1998–2000 men and women from EPIC, now aged 42 to 82 years, were invited to attend a second visit where measurements of QUS of the heel were conducted by trained nurses working to standard protocols. Both speed of sound (SOS) and broadband ultrasound attenuation (BUA) were recorded for each subject. Measurement was made using a CUBA sonometer (McCue Ultrasonics, Winchester) at least twice on each foot. The mean of the measures (left and right foot) were used for analysis. The coefficient of variation was 3.5% [16]. Five CUBA machines were used and each was calibrated daily with its physical phantom. A roving physical phantom was used monthly to check calibration between machines and room temperature measured daily.

### Analysis

For each individual notified to NOAR who had had heel ultrasound measurements performed, two age and sex matched controls were selected from EPIC-Norfolk. The analysis was divided into two stages. In the first, fixed effect linear regression was used to look at the association between heel ultrasound measurement and case status, with the bone ultrasound parameter (SOS or BUA) as the outcome and case status as the independent variable. As our aim was to examine the influence of IP on bone health, this approach allowed us to quantify the association in terms of the mean absolute difference (and 95% confidence interval) in the bone ultrasound parameters between the cases and controls which we felt was easy to interpret. Adjustments were subsequently made for body mass index and also steroid therapy (as assessed in EPIC-Norfolk). In the second stage we used simple linear regression to examine the influence of the following disease related factors assessed at baseline on the ultrasound parameters : swollen and tender joint count, HAQ, rheumatoid factor, and whether the subject satisfied the 1987 ACR criteria at baseline (both the traditional four of seven criteria and the decision tree approach [34] and the disease duration as determined from the time of symptom onset until the ultrasound scan, after adjustment for age and sex [34]. For the purposes of analysis, the joint counts were categorised into tertiles, the lowest tertile representing those with the least number of involved joints. The results were expressed as β coefficients and 95% confidence intervals (CI). Having determined the disease related predictors of the heel ultrasound parameters, we then used fixed effect linear regression to determine whether or not these explained differences between those with IP and the non IP controls. Statistical analysis was performed using STATA (Stata Corporation. Stata Statistical Software: Release 9.2, College Station, TX, USA, 2008).

## Results

### Subject characteristics

In all, 139 subjects (52 men and 87 women) with IP and 278 age and sex matched controls were studied. The characteristics of these subjects are presented in Table 1. Women with IP had a slightly greater BMI (26.9 kg/m^2^ vs 25.6 kg/m^2^) and (as expected) a higher prevalence of steroid use (17.2% vs 1.7%) than their matched controls. In men, there were no differences between those with and without IP with respect to body mass index or steroid use.

**Table 1:** Subject characteristics

**Variable**	**Men**			**Women**		
	**IP**	**Non-IP**		**IP**	**Non-IP**	
	**N = 52**	**N = 104**		**N = 87**	**N = 174**	
	**mean (SD)**	**mean (SD)**	**p value**	**mean (SD)**	**mean (SD)**	**p value**
Age at interview (years)	65.7 (8.1)			61.7 (9.0)		
Body mass index at baseline (kg/m^2^)	26.9 (3.4)	26.6 (3.4)	0.68	26.9 (4.3)	25.6 (4.0)	0.02
	%	%		%	%	
Steroid tablets/injections for at least 3 months	5.8	4.8	0.80	17.2	1.7	<0.001

IP = inflammatory polyarthritis.

Disease related characteristics at baseline are presented in Table 2. The mean duration of disease from symptom onset to time of ultrasound was 4.8 years (standard deviation [SD] = 2.9) in men and 5.6 years (SD = 2.8) in women (mean duration of symptoms prior to baseline assessment in NOAR was 0.9 years (SD = 1.1) in men and 1.3 years (SD = 1.9) in women). In terms of joint involvement, men had a median of 1.0 (interquartile range [IQR] = 0.0,3.5) and women a median of 2.0 (IQR = 0.0, 5.0) joints that were both swollen and tender. The median HAQ score was 0.5 (IQR = 0.1, 1.1) in men and 0.8 (IQR = 0.4, 1.3) in women. 39% of men and 34% of women were rheumatoid factor positive and 42% and 60% respectively satisfied the ACR criteria for rheumatoid arthritis using the tree definition. Given the design (see methods) only 27 subjects had hand radiographs at baseline. Of these 75% of men and 56.5% of women had evidence of erosive disease.

**Table 2:** Disease characteristics of subjects with IP at baseline

**Variable**	**Men N = 52**	**Women N = 87**
	**Mean (SD)**	**Mean (SD)**
Duration of arthritis^*^ (years)	4.8 (2.9)	5.6 (2.8)
	Median (IQR)	Median (IQR)
Number of swollen joints	2.0 (1.0, 8.0)	5.0 (2.0, 11.0)
Number of tender joints	5.0 (2.0, 8.5)	6.0 (2.0, 15.0)
Number of both swollen and tender joints	1.0 (0.0, 3.5)	2.0 (0.0, 5.0)
Larsen score^1^	27.5 (13.0,31.0)	11.0 (1.0, 21.0)
HAQ score (0–3)	0.5 (0.1, 1.1)	0.8 (0.4, 1.3)
	%	%
Rheumatoid factor + ve	39.1	33.8
Erosions^1^	75.0	56.5
Satisfied ARA RA criteria (4/7 definition)	22.9	45.8
Satisfied ARA RA criteria (tree definition)	41.7	60.2
Steroid use for at least 3 months	5.8	17.2

^*^Years between first symptom onset and ultrasound scan.

^1^Assessed in those with baseline radiographs (n = 27).

### Influence of IP on SOS and BUA

Overall, mean SOS was 1621.8 m/s (SD = 43.4) and BUA was 76.3 dB/MHz (SD = 18.4) in those with IP (Table 3). Mean SOS was lower in those with IP than the controls for both men (1629.0 vs 1644.0 m/s) and women (1617.6 vs 1624.5 m/s) though the difference was statistically significant for men only. There was no difference in BUA between those with IP and their matched controls in men or women. There was no evidence of interaction between sex and case status, BMI or steroid use and in subsequent analysis the data for men and women were pooled. Using fixed effect linear regression, with the bone ultrasound parameter as the dependent variable and case status as the independent variable, SOS was lower among those with IP than the matched controls (difference −10.0; 95%CI −17.4, -2.6), a difference of 0.6%, see Table 4. This difference persisted after adjusting for body mass index and steroid use (difference −9.6; 95%CI −17.9, -1.3). BUA, however, was similar between those with IP and controls (difference −1.2; 95%CI −4.5, 2.1). When the analysis was restricted to those who fulfilled the ACR tree criteria at baseline the differences between those with IP and the controls became more marked for both SOS (difference = −17.3; 95% CI −28.8, -5.7) and for BUA, which now became statistically significant (difference = −7.2;95% CI −12.4, -2.1). Higher BMI at baseline was associated with a higher BUA (β coefficient 1.1; 95%CI 0.6, 1.6) independent of case status. BMI and steroid use were not independently associated with SOS, see Table 4.

**Table 3:** Ultrasound characteristics

**Variable**	**Men**			**Women**		
	**IP**	**Non-IP**		**IP**	**Non-IP**	
	**N = 52**	**N = 104**		**N = 87**	**N = 174**	
	**mean (SD)**	**mean (SD)**	**p value**	**mean (SD)**	**mean (SD)**	**p value**
Broadband ultrasound attenuation (dB/MHz)	85.8 (18.1)	91.1 (18.6)	0.09	70.6 (16.2)	69.3 (14.7)	0.52
Speed of sound (m/s)	1629.0 (47.6)	1644.0 (35.5)	0.03	1617.6 (40.3)	1624.5 (38.9)	0.18

IP = inflammatory polyarthritis.

**Table 4:** Influence of case/control status, body mass index (BMI) and steroid use on ultrasound parameters in men and women

	**Broadband attenuation (dB/MHz)**		**Speed of sound (m/s)**	
	**Univariate**^**a**^	**Multivariate**^**b**^	**Univariate**^**a**^	**Multivariate**^**b**^
	**β coeff (95% Cl)**	**β coeff (95% Cl)**	**β coeff (95% Cl)**	**β coeff (95% Cl)**
Case/Control status				
control	Referent	Referent	Referent	Referent
case	−1.2 (−4.5, 2.1)	−2.6 (−6.1, 1.0)	−10.0 (−17.4, -2.6)*	−9.6 (−17.9, -1.3)*
BMI at baseline (kg/m^2^)	1.1 (0.6, 1.6)*	1.1 (0.6, 1.6)*	0.6 (−0.6, 1.8)	0.8 (−0.4, 2.0)
Steroids ≥3 months				
no	Referent	Referent	Referent	Referent
yes	6.1 (−1.6, 13.7)	6.8 (−1.7, 15.3)	−2.2 (−19.9, 15.5)	1.5 (−18.3, 21.3)

^a^unadjusted model.

^b^model includes case/control status, BMI at baseline and steroid use.

*p < 0.05.

### Influence of disease related factors on SOS and BUA

The influence of disease related factors on ultrasound parameters is presented in Table 5. After adjusting for age and sex, compared to those in the lowest tertile of swollen and tender joint counts, those in the mid and upper tertile had lower BUA though this was significant for the middle tertile only (difference = −6.4; 95%CI −12.7, -0.2). Compared to those who did not satisfy the ACR criteria (either list or tree definition) those that did satisfy the criteria had a lower BUA (difference = −8.5; 95%CI −13.7, -3.2 using the tree definition). With respect to SOS, compared to those in the lowest tertile of swollen joint count, those in the mid and upper tertiles had a lower SOS which was significant for the upper tertile (difference = −20.1; 95%CI −35.6, -4.6). There was evidence also of a significant linear trend for an increase in both the swollen joint count and swollen and tender joint count and lower SOS. Compared to those who did not satisfy the ACR criteria (using either list or tree definition), those that did also had a reduced SOS (difference = −18.7; 95%CI −32.1, -5.2 using the tree definition). Larsen score, presence of erosions (in those who had X-rays performed at baseline), disease duration (time between symptom onset and the ultrasound scan), rheumatoid factor and HAQ were not associated with the ultrasound parameters.

**Table 5:** Influence of IP baseline characteristics on ultrasound parameters in men and women

	**Broadband attenuation**	**Speed of sound**
	**(dB/MHz)**	**(m/s)**
	**βcoeff (95% Cl)**^**a**^	**βcoeff (95% Cl)**^**a**^
Symptom duration^1^ (years)	0.3 (−0.7, 1.2)	−0.7 (−3.2, 1.7)
Rheumatoid Factor+		
no	Referent	Referent
yes	−4.0 (−9.8, 1.9)	−4.9 (−19.7, 10.0)
Tertiles of swollen joint count		
low	Referent	Referent^b^
mid	−4.1 (−10.8, 2.7)	−6.3 (−23.2, 10.7)
high	−6.0 (−12.2, 0.1)	−20.1 (−35.6, -4.6)*
Tertiles of tender joint count		
low	Referent	Referent
mid	2.4 (−4.3, 9.1)	−4.3 (−21.1, 12.6)
high	−1.9 (−8.5, 4.7)	−13.3 (−29.9, 3.3)
Tertiles of both swollen & tender joint count		
low	Referent	Referent^b^
mid	−6.4 (−12.7, -0.2)*	−13.7 (−29.6, 2.2)
high	−5.3 (−12.0, 1.4)	−16.1 (−33.1, 0.9)
Larsen score^2^	0.1 (−0.2, 0.3)	−0.1 (−1.0, 0.7)
Erosions^2^		
no	Referent	Referent
yes	0.9 (−8.3, 10.0)	8.2 (−23.3, 39.7)
Satisfied ARA criteria (4/7 definition)		
no	Referent	Referent
yes	−7.8 (−13.4, -2.3)*	−18.9 (−33.1, -4.8)*
Satisfied ARA criteria (tree definition)		
no	Referent	Referent
yes	−8.5 (−13.7, -3.2)*	−18.7 (−32.1, -5.2)*
HAQ score	−0.6 (−5.4, 4.3)	−2.1 (−14.5, 10.3)
Steroids ≥3 months		
no	Referent	Referent
yes	7.3 (−0.7, 15.3)	9.7 (−11.1, 30.6)

^a^adjusted for age and sex; ^b^p < 0.05 test for trend; *p < 0.05.

^1^Years between first symptom onset and ultrasound scan.

^2^Assessed in those with baseline radiographs (n = 27).

We looked next at whether the swollen joint count as a marker of inflammation could explain the difference between cases and controls, assuming that the controls had no inflamed joints. After adjusting for BMI, steroid use and number of swollen joints the difference in SOS between those with and without IP became much weaker and non significant (difference = −3.9; 95%CI −14.6, 6.7).

## Discussion

In this analysis, subjects with IP presenting to primary care had a modestly but significantly lower SOS though not BUA compared to population based controls. The effect was more marked among those who satisfied ACR criteria at baseline and for these subjects BUA was also lower than controls. Disease activity as determined by the swollen joint count explained in part the reduction in SOS in women with IP.

Our study has several advantages: it was population based with both cases and controls nested within the population-derived cohort. It used standard methods in assessment of both IP and also assessment of heel ultrasound. There are though several limitations which need to be considered when interpreting the results. Not all patients with an episode of IP in the Norfolk area will have presented to primary care and among those that do it is likely that not all will have been referred or notified to NOAR. Such patients are likely to have less severe disease than those reported to NOAR and thus the study is likely to have missed some subjects with mild disease. All of the joint assessments were undertaken by one of a team of trained research nurses which may have introduced error into measures such as the joint count. Any imprecision in assessment would tend to attenuate significant biological associations. The ultrasound assessments were also undertaken at a variable duration after the onset of the arthritis. The average disease duration was short at around 5 years until the time of the scan, however, duration of disease had no effect on the results. In this regard it is interesting to note that a one off baseline joint assessment seemed to explain most of the difference, albeit quantitatively small, in SOS between cases and controls. One interpretation of this finding is that any influence of disease is predicted from the earliest stage. There wasn’t sufficient information to examine the influence of diagnoses other than RA. There was also no formal assessment of physical activity at baseline and it was not possible therefore to look at the effect of this on the bone parameters subsequently assessed. Finally, our results relate to a cohort of European Caucasian men and women living in the Norfolk (UK) area, so caution is required in extrapolation beyond this group.

How do our results compare with previous studies? Our data in relation to subjects with IP is consistent with previous studies which suggest a reduction in SOS among patients with RA (based on ACR criteria) compared to non RA controls though the reduction (0.6%) is less marked than observed in most of these studies [22-24,26,28-30]. In contrast to previous studies in RA patients we found no association between IP and BUA, though among those subjects in our study who satisfied the ACR criteria (tree definition) at baseline, BUA was reduced compared to age matched controls (70.6 vs 77.5 dB/MHz). To our knowledge there are no other data concerning the influence of undifferentiated inflammatory polyarthritis on heel ultrasound parameters.

Data concerning the impact of disease related variables on heel ultrasound parameters are somewhat conflicting. Some, though not all, report an association between SOS and BUA with disease activity as assessed by ESR, CRP or swollen/tender joint counts [26-29]. In addition some reports suggest an association with the HAQ score [22,24-29,31]. Our data support an association between disease activity as determined by swollen joint and a combined swollen and tender joint and SOS. We found no association, however, with HAQ score and no association with other disease related parameters including disease duration or rheumatoid factor.

Studies in men and women suggest that both SOS and BUA are important predictors of fracture [15-21]. The mechanism by which IP might influence these parameters is unclear though it is possible that increased bone turnover perhaps related to increased cytokine production in those with active disease may influence bone [36-38]. Our finding of an association between SOS and the swollen joint count would be consistent with this.

To date there are no data from prospective studies relating bone ultrasound parameters in RA or IP to fracture risk and therefore the extent to which the results are clinically relevant is as yet unclear. There is some evidence from cross-sectional studies that BUA and SOS are lower in RA patients with vertebral deformity than those without [25,30]. Prospective studies are, however, required including assessment of ultrasound, measurement of bone mass and collection of fracture data in order to determine the clinical relevance of ultrasound in IP.

## Conclusions

In conclusion in this primary-care derived cohort of patients with inflammatory polyarthritis there is evidence from ultrasound of a potentially adverse effect on the skeleton. The effect appears more marked in those with active disease.

## Competing interests

The authors declare that there are no competing interests.

## Authors’ contributions

SP performed the statistical analysis and drafted the manuscript. TM participated in the design and data collection of the study and contributed to the drafting of the manuscript. KG participated in the design and data collection of the study and contributed to the drafting of the manuscript. RL participated in the design and data collection of the study and contributed to the drafting of the manuscript. KTK participated in the design and data collection of the study and contributed to the drafting of the manuscript. AJS conceived the study, participated in its design and coordination and helped to draft the manuscript. DPMS conceived the study, participated in its design and coordination and helped to draft the manuscript. TWO conceived the study, participated in its design and coordination and helped to draft the manuscript. All authors read and approved the final manuscript.

## Pre-publication history

The pre-publication history for this paper can be accessed here:

http://www.biomedcentral.com/1471-2474/13/133/prepub
